# To Share or Not to Share? The Role of Retailer’s Information Sharing in a Closed-Loop Supply Chain

**DOI:** 10.3389/fpsyg.2022.941952

**Published:** 2022-07-18

**Authors:** Huaige Zhang, Xianpei Hong, Xinlu Cao

**Affiliations:** ^1^School of Business Administration, Guangdong University of Finance and Economics, Guangzhou, China; ^2^School of Business Administration, South China University of Technology, Guangzhou, China

**Keywords:** supply chain management, information sharing, remanufacturing, dual recycling channels, power structure

## Abstract

Retailers are faced with a dilemma of whether to share demand information with other supply chain members, and if so, how to share it. Our research interest is motivated by the grounds that the value of downstream retailers’ sales information to upstream manufacturers is to improve the accuracy of manufacturers’ order forecasting. This problem is particularly important in the remanufacturing of closed-loop supply chains (CLSCs). In this study, we consider a retailer (she) as the demand information holder, who sells new and remanufactured products in wholesale to a manufacturer (he) and, simultaneously, she and the manufacturer competitively collect used products from the customers. We explicitly characterize the role of information sharing in a CLSC. We contributed to the information-sharing literature by integrating the existing information-sharing model with dual recycling channels and channel power structure. Previous literature suggests that retailers prefer to share demand information with other firms when the market demand is high. However, surprisingly, we find that when the manufacturer does not play a leading role, the retailer shares her forecast demand information with the manufacturer if the market demand is low. We also show that information sharing reduces the overall profit of the supply chain when the manufacturer dominates the market. In addition, our results also illustrate that information sharing affects the performance of the supply chain mainly by affecting the wholesale price.

## Introduction

Information sharing among members can enhance the coordination of the supply chain with the advances in information technology (Hosoda et al., 2015; Zhou et al., 2017; Li et al., 2021; Jain, 2022). Our research interest is motivated by the grounds that the value of downstream retailers’ sales information to upstream manufacturers is to improve the accuracy of manufacturers’ order forecasting, according to some empirical and theoretical researches on the value of information sharing. Better prediction accuracy may lead to lower safety inventory and better service for manufacturers (Cachon and Fisher, 2000; Chen et al., 2000; Chen and Lee, 2009). Cui et al. (2015) show that the improvement in the mean square forecast error for all studied products ranged from 7.1 to 81.1% if the company included the retailer’s sales data into the demand forecast based on the data collected from a consumer packaging company. In recent years, remanufacturing of closed-loop supply chains (CLSCs) has made a dent in industry and academia due to the scarcity of material resources (Govindana et al., 2015; Liu B. Y. et al., 2019; Aminipour et al., 2021; Qiao and Su, 2021; Soleimani et al., 2021; Zhang and Zhang, 2022). Although many studies have shown that remanufacturing can reduce production costs by 40–65%, Akçalı and Çetinkaya (2011) argue that compared to the traditional supply chain, the coordination problem is more sophisticated due to the uncertainty of the recycle rate and market demand in the CLSC.

Although many studies look at the retailer’s decision about sharing demand information in the traditional supply chain (Li and Zhang, 2008), academic researches still ignore some gaps regarding information-sharing demand. Previous studies ignore the influence of dual recycling channels on the retailer’s decision with regard to demand information sharing under different channel power structures. As far as we know, the retailer’s decision on whether to share forecast demand information is very important for profits. When the retailer is the leader, she has more control over her information-sharing decision. In addition, compared with a single recycling channel, dual recycling channels have higher efficiency (Hong et al., 2013; Huang et al., 2013).

To bridge these gaps, we further expand the influence of dual recycling channels on the retailer’s information-sharing decision in the CLSC under three power structures. We construct a CLSC model that considers three elements of information sharing, channel power structure, and dual recycling channels.

To answer the research question, we present a two-echelon game model to compare and analyze the supply chain members’ optimal decisions to promote supply chain coordination under three-channel power structures (Jin et al., 2021). We assume that the two-echelon model has only two participants, the manufacturer and the retailer. The manufacturer sells new products and remanufactured products through the retailer. The manufacturer and retailer synchronously recycle used products from consumers and compete with each other. The retailer can forecast customer’s demand information and has the right to determine whether to share forecast demand information. This study analyses the optimal retailer’s decision about demand information sharing when the manufacturer dominates the market, the retailer dominates the market, and the retailer and the manufacturer are equally matched in the market (i.e., there is no leader in the market).

This study provides several theoretical contributions and practical implications. First, we make a contribution to the information-sharing literature by integrating the existing information-sharing model with dual recycling channels and channel power structure. Second, we find some interesting results. When the manufacturer and the retailer compete to recycle used products, the retailer conceals her forecast demand information if the manufacturer dominates the market; the retailer shares her demand information with the manufacturer if the market demand is low, and the retailer plays a dominant role in the market. In the Nash game between the manufacturer and the retailer, the situation is similar to when the retailer is the leader in the market. Finally, this study provides some valuable insights into the retailer’s information-sharing decision. Information always is an important influencing factor for enterprises to make decisions. However, in reality, it is difficult for enterprises to obtain complete market information because when enterprises have private information, they can bring additional benefits; enterprises generally conceal their proprietary information from other participants. Motivating participants to share demand information is an important means to enhance supply chain performance.

The rest of this study is organized as follows. We review the related literature about information sharing, dual recycling channels, and channel power structure in section “Literature Review.” We present our assumes and model in sections “Descriptions” and “Model Framework.” We analyze how dual recycling channels affect the retailer’s information-sharing decision under the three power structures in sections “Comparison and Analysis of Results” and “The Value of Information Sharing.” Finally, we give the conclusion in section “Numerical Examples.” We put all the proofs in the Supplementary Appendix.

## Literature Review

Our study relates to the literature on three dimensions: the literature on information sharing, the literature on dual recycling channels, and the literature on the channel power structure, each of which we review below.

The first stream is about information sharing. Many studies have explored the role of information sharing in the positive channel (Gal-Or et al., 2008; Li and Zhang, 2008; Chen and Lee, 2009; Shamir and Shin, 2015; Shang et al., 2016; Huang et al., 2018). They argue that retailers can induce manufacturers to cut wholesale prices by disclosing low demand and withholding high demand. Li (2002) considers that information sharing brings both “direct effects” and “indirect effects” to the manufacturer. The above researches focus on what is the retailer’s condition for sharing her private information with other participants (Zhang Q. et al., 2019). A few studies have shown that manufacturers as participants in the supply chain can also share information in some cases where the manufacturer possesses better demand information than the downstream retailer (Jiang et al., 2016; Zhou et al., 2017; He et al., 2018). In addition, information sharing may occur between retailers and consumers (Liu Y. et al., 2019). Absolutely, some studies look at information sharing within an enterprise where the sales department is responsible for forecasting demand and the operations department is responsible for ordering (Scheele et al., 2018). An increasing number of studies are paying attention to information sharing in dual channels (Ha and Tong, 2008; Guo et al., 2014; Ha et al., 2017). Information sharing is beneficial to the supply chain, and the dominant strategy is to reduce the investment cost of information sharing in the supply chain with information sharing. A supply chain without information sharing has lower product sales by comparing the performance of the two types of supply chains (i.e., supply chains with and without information sharing). Existing researches focus on how information sharing enhances the product sales in the traditional supply chain (Zhang and Zhang, 2020). Unlike existing studies, we consider how dual recycling channels affect the retailer’s information-sharing decision in the CLSC.

The second stream is about dual recycling channels. Although the dual-channel problem has been mentioned frequently, most of the relevant studies focus on sales in dual forward channels; the following part discusses the application of dual channels in a CLSC (Li and Zhang, 2008; Bandyopadhyay and Paul, 2010; Ma et al., 2013; Feng et al., 2017; He et al., 2019). The manufacturer always faces the challenge of strategically designing the reverse recycling channel because the price competition is between two channels (Feng et al., 2017; Zhang S. G. et al., 2019; Shekarian et al., 2021). Most studies about dual recycling channels concern recycling by supply chain participants, such as manufacturers or retailers (Hong et al., 2013; Huang et al., 2013; Liu et al., 2017). Bulmus et al. (2014) study the recycling competition between an OEM and a third-party remanufacturer in a two-period closed-loop supply chain model. Some studies divide recycling channels into formal and informal recycling channels (Liu et al., 2016). As for dual-channel researches, most researches focus on how manufacturers design and choose the power channel to sell their products in the positive channel. However, only a few studies investigate the influence of dual recycling channels on the retailers’ information-sharing decision on CLSCs (Lei et al., 2014). Few studies consider the interaction between information-sharing decision and dual recycling channels. Existing studies on remanufacturing primarily consider distribution channels and marketing competition (Shi et al., 2020; Nie et al., 2021). Apart from existing studies, we consider recycling competition in a double-recycling channel CLSC. We look at how the manufacturer can achieve efficient recycle of used products and how the retailer’s information-sharing decision affects the recycle of used products different from the existing literature.

The third stream is about the channel power structure. The “power of the supply chain member” receives plentiful attention from academia in the CLSC, enterprises as well as in the mass media. Kadiyali et al. (2000) explains power in his research: The power is based on the proportion of channel profits obtained by each channel member. Power structure has attracted a lot of attention because the firm that has higher channel power gains more profit (Majumder and Srinivasan, 2008; Pan et al., 2010; Shi et al., 2013; Chen and Wang, 2015). Most of the existing literatures consider the market structure where the manufacturer dominates the market but ignore the market power of retailers and third-party recyclers (Savaskan et al., 2004; Raju and Zhang, 2005; Savaskan and Wassenhove, 2006; Chen et al., 2012). However, many examples of enterprises prove that there are strong retailers as the leader or the retailer and manufacturer are evenly matched in the market (Edirisinghe et al., 2011; Xiao et al., 2014; Luo et al., 2017). The influence of information sharing on dual channels is different under different power structures (Ha et al., 2011). There have been many researches on information sharing, dual recycling channels, and channel power structure in the CLSC, but only a few studies on the coordination among the three elements (Yue and Liu, 2006; Huang and Wang, 2017). Unlike previous studies that considered manufacturers usually playing a leading role in the market (Vedantam and Iyer, 2021), our research focuses on different channel power structures. Given the lack of the influence of different channel power structures on the information-sharing decision, we study how to make an optimal information-sharing decision under different power structures when the manufacturer and retailer synchronously recycle used products.

Based on the above considerations, this study structures a CLSC model that considers three elements of information sharing, channel power structure, and dual recycling channels, and studies the problems such as formulating pricing strategies, designing coordination contracts, and optimizing system efficiency in the CLSC.

## Descriptions

**FIGURE 1:**
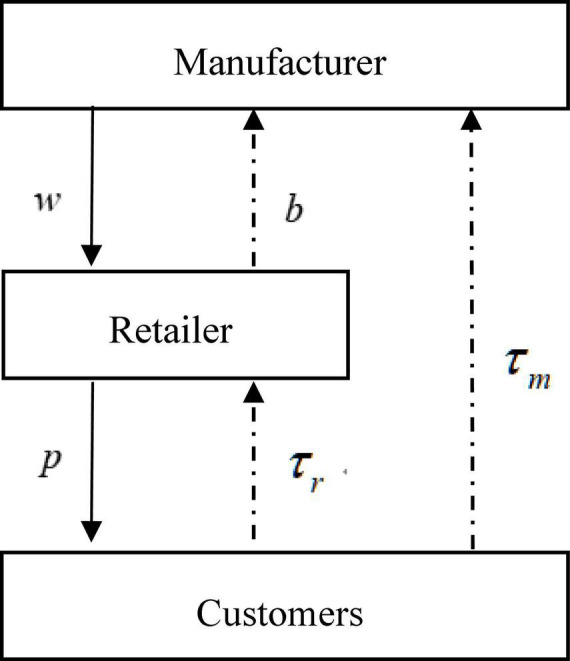
The model structure diagram of this study.

We structure a CLSC system that only has a monopoly manufacturer and a monopoly retailer in the market (Jin et al., 2021). As shown in Figure 1, the retailer purchases new and remanufactured products from the manufacturer at wholesale price *w*, and then sells products to customers at retailer price *p* in the forward channel. We assume that the retailer can predict the demand and has the right to decide whether to share this demand information. In the reverse channel, consumers can choose to recycle used products through the manufacturer or the retailer. In addition, the manufacturer recycles used products from the retailer by paying the transfer fee *b* and undertakes the remanufacturing operation.

We show the parameters involved in this study and their meanings in Table 1. $\pi_{i}^{j}$ represents the profit of member *i* in the supply chain in model *j*. The superscript *j* ∈ {*M*−*S*, *M*−*N*, *R*−*S*, *R*−*N*, *M*−*R*−*S*, *M*−*R*−*N*} represent, respectively, the Stackelberg model that the manufacturer dominates the market with and without information sharing, the Stackelberg game model that the retailer dominates the market with and without information sharing, and the Nash model between the manufacturer and the retailer with and without information sharing. The subscript *i* ∈ {*m*, *r*, *s*} represent, respectively, the manufacturer, the retailer and the supply chain system. $V_{i}^{l}$ represents in model *l* the information value that information-sharing behavior of retail brings to supply chain participants *i*. The superscript *l* ∈ {*M*, *R*, *M*−*R*} represent, respectively, the Stackelberg model that the manufacturer dominates the market, the Stackelberg model that the retailer dominates the market, and the Nash model between the manufacturer and the retailer.

**TABLE 1:** The description of the symbols.

Symbol	Descriptions
α	Potential market size
β	Coefficient of price elasticity
*w*	Wholesale price
*p*	Retailer price
*c*_*m*_	Unit production cost of new product
*c*_*r*_	Unit production cost of remanufactured product
*b*	The transfer price paid by the manufacturer to the retailer
τ_*r*_	Retailer’s recycling rate
τ_*m*_	The manufacturer’s recycling rate
*a*	Recycle competition intensity
$\pi_{i}^{j}$	The profit of member *i* of the supply chain in model *j*

We assume that the manufacturer produces new and remanufactured products (Nie et al., 2021). The manufacturer undertakes not only the business of directly using raw materials to produce new products, but also the business of remanufacturing used products. The unit production cost of a product made from old recycled products is lower than the unit production cost of a new product (i.e., *c*_*m*_ > *c*_*r*_) (Jin et al., 2022). We make Δ = *c*_*m*_−*c*_*r*_ and Δ > 0, which means the unit production cost saved by the manufacturer in producing remanufactured products from the recycled used products. According to the research of Savaskan et al. (2004), we consider remanufactured products are indistinguishable from new products in terms of quality and function. They are invested in the market at the same retail price through the same channels. Consumers are equally willing to pay for new and remanufactured products (Huang et al., 2013). We assume that the market demand is *D* = α−β*p*, where α is the potential market demand, β is the price elasticity coefficient, α > 0, β > 0, α > β*c*_*m*_ (Kushwaha et al., 2022). Note that this linear demand function is widely used in remanufacturing CLSC literature (Rahmani et al., 2020; Zhou et al., 2021).

We consider that the manufacturer and retailer synchronously recycle used products. In this study, *a* represents the recycling competition intensity between the manufacturer and the retailer. The larger the *a* is, the more competitive the recycle is, and 0 ≤ *a* ≤ 1. Huang et al. (2013) have the same hypothesis that competition affects each other symmetrically. Consistent with Hong et al. (2017), we use the cost structure in Eq. (1) to describe the manufacturer and retailer recycling rates:


(1) τm=Im-a⁢IrCL,τr=Ir-a⁢ImCL,0≤τ+mτr≤1,0≤a≤1

Where, *I*_*m*_ and *I*_*r*_ represent, respectively, the fixed investment of the manufacture and the retailer in recycling used products. *C*_*L*_ is a scale parameter and assumed enough to ensure τ_*T*_ < 1, where τ_*T*_ represents the total recycling rate.

The potential market demand α is a random variable, i.e., α = α_0_ + *e*, where, α_0_ is the part of the market where potential demand is determined. *e* is the uncertain part of market demand caused by indefinite elements. The expectation of random variable *e* is 0 and the variance is *k* (Ha et al., 2014).

Because the retailer is closer to customers than the manufacturer in the supply chain structure, she can use her structural advantages to predict the uncertain part of the market demand. The prediction of market information by the retailer can help supply chain participants make decisions. A large number of studies have made similar assumptions, such as Huang and Wang (2017). We set the market demand predicted by the retailer is *f*, and *f* = α + ε, where ε is the error term whose expectation is 0 and variance is θ. Random variables *e* and ε are independent of each other. According to the research of Li (2002), we have the information structure assumption of Eq. (2):


(2) $$E\,\left(\alpha |f\right)=\frac{\theta }{k+\theta }\,\alpha_{0}+\frac{k}{k+\theta }\,f\equiv A,E\,\left({\left(f-\alpha_{0}\right)}^{2}\right)=k+\theta$$

We assume that *t* represents the accuracy of the retailer’s prediction of market demand and *t* ∈ (0,1). There are two extremes: when *t* = 0, the predicted value of the market demand by the retailer is completely different from actual market demand. At this time, the accuracy of the prediction of the market demand by the retailer is the lowest. When *t* = 1, the predicted market demand by the retailer is exactly the same as the actual market demand. At this time, the accuracy of predicted market demand by retailer is the highest.

In our study, the manufacturer pays the retailer a transfer fee *b* when collecting used products from the retailer, where 0 ≤ *b* ≤ Δ. In order to simplify the derivation of the model, we assume that the fee for paying to consumers is zero, which has no change on the conclusion of our study. Savaskan and Wassenhove (2006) adopt the same assumption. Finally, we assume that products are produced according to orders, so the manufacturer and the retailer have no inventory cost when selling products (Li, 2002).

## Model Framework

This study considers that retailer has two options for the private demand information: (1) share demand information with the manufacturer and (2) withholding demand information from the manufacturer. Next, we consider three power structures with/without information sharing about customers’ demand, namely the manufacture as a leader (Model M), the retailer as a leader (Model R), and the Nash game (Model M–R) under this assumption.

If the retailer hides her forecast demand information from the manufacturer, the manufacturer makes decisions only based on the determined part of market information, while the retailer makes decisions based on her own forecast of market demand. We describe the optimal expected profits of manufacturer and retailer as follows [Eqs. (3, 4)]:


(3) $$\begin{array}{c} \underset{p,\tau_{r}}{M\,a\,x}E\left(\pi_{r}|f\right)=(\left(p-w\right)\left(\alpha -\beta p\right)+b\tau_{r}\left(\alpha -\beta p\right)-\\ \frac{C_{L}\,\left(\tau_{r}^{2}+a\,\tau_{m}^{2}\right)}{1-a^{2}}|f) \end{array}$$

(4) $$\begin{array}{c} \underset{w,\tau_{m},b}{M\,a\,x}E\left(\pi_{m}\right)=[\left(w-c_{m}+\Delta \left(\tau_{m}+\tau_{r}\right)\right]\left(\alpha -\beta p\right)-\\ \frac{C_{L}\,\left(\tau_{m}^{2}+a\,\tau_{r}^{2}\right)}{1-a^{2}}-b\,\tau_{r}\,\left(\alpha -\beta \,p\right) \end{array}$$

The manufacturer and retailer both make optimal decisions according to demand information by predicting if the retailer shares demand information. The target function of the retailer is calculated as per Eq. (3), and the manufacturer’s expected profit decision model is as follows [Eq. (5)]:


(5) $$\begin{array}{c} \underset{w,\tau_{m},b}{M\,a\,x}E\left(\pi_{m}^{M-S}|f\right)=([\left(w-c_{m}+\Delta \left(\tau_{m}+\tau_{r}\right)\right]\left(\alpha -\beta p\right)-\\ \frac{C_{L}\,\left(\tau_{m}^{2}+a\,\tau_{r}^{2}\right)}{1-a^{2}}-b\tau_{r}\left(\alpha -\beta p\right)|f) \end{array}$$

### Model M

Manufacturers usually play a leading role in the market based on real business examples (Vedantam and Iyer, 2021). In this case, the manufacturer occupies the dominant position in the market and can make decisions in the game process in priority and according to the reaction of the retailer. As a follower of the market, the retailer makes decisions according to the decisions of the manufacturer. When selling products, the retailer can only determine the retail price of products according to the wholesale price determined by the manufacturer. The manufacturer and retailer synchronously recycle used products. The manufacturer reproduces the used products and puts them and new products in the market for sale.

At this point, we set the game sequence of the model as follows:

(1) The manufacturer first determines the wholesale price *w* of new and remanufactured products, the manufacturer’s recycling rate τ_*m*_ for used products, and the transfer price *b* to the retailer.
(2) The retailer sets the retail price *p* of new and remanufactured products and the retailer’s recycling rate τ_*r*_ for used products.

**Propositions 1:** When the manufacturer is the market leader without information sharing, the optimal decisions of the manufacturer and retailer are as follows:


$$w^{M-N^{*}}=\frac{\left[4\,C_{L}-\beta \,\Delta^{2}\,\left(1-a^{2}\right)\,\left(2-a\right)\right]\,\alpha_{0}+\left[4\,C_{L}-\beta \,\Delta^{2}\,\left(1-a^{2}\right)\right]\,\beta \,c_{m}}{\beta \,\left[8\,C_{L}-\beta \,\Delta^{2}\,\left(1-a^{2}\right)\,\left(3-a\right)\right]},$$

$$\begin{array}{c} p^{M-N^{*}}=\frac{\left[6\,C_{L}-\beta \,\Delta^{2}\,\left(1-a^{2}\right)\,\left(3-a\right)\right]\,\alpha_{0}+2\,C_{L}\,\beta \,c_{m}}{\beta \,\left[8\,C_{L}-\beta \,\Delta^{2}\,\left(1-a^{2}\right)\,\left(3-a\right)\right]}+\\ \frac{\left[6\,C_{L}-\beta \,\Delta^{2}\,\left(1-a^{2}\right)\,\left(3-a\right)\right]\,t\,\left(f-\alpha_{0}\right)}{\beta \,\left[8\,C_{L}-\beta \,\Delta^{2}\,\left(1-a^{2}\right)\,\left(3-a\right)\right]}, \end{array}$$

$\tau_{m}^{M-N^{*}}=\frac{\Delta \,\left(1-a^{2}\right)\,\left(\alpha_{0}-\beta \,c_{m}\right)}{8\,C_{L}-\beta \,\Delta^{2}\,\left(1-a^{2}\right)\,\left(3-a\right)}$ and $\tau_{r}^{M-N^{*}}=\frac{\Delta \,\left(1-a^{2}\right)\,\left(\alpha_{0}-\beta \,c_{m}\right)}{8\,C_{L}-\beta \,\Delta^{2}\,\left(1-a^{2}\right)\,\left(3-a\right)}+$$\frac{\Delta \,\left(1-a^{2}\right)\,t\,\left(f-\alpha_{0}\right)}{8\,C_{L}-\beta \,\Delta^{2}\,\left(1-a^{2}\right)\,\left(3-a\right)}$

**Propositions 2:** When the manufacturer is the market leader with information sharing, the optimal decisions of the manufacturer and retailer are as follows:


$$w^{M-S^{*}}=\frac{\,\left[4\,C_{L}-\beta \,\Delta^{2}\,\left(1-a^{2}\right)\,\left(2-a\right)\right]\,\left[\alpha_{0}+t\,\left(f-\alpha_{0}\right)\right]+\left[4\,C_{L}-\beta \,\Delta^{2}\,\left(1-a^{2}\right)\right]\,\beta \,c_{m}}{\beta \,\left[8\,C_{L}-\beta \,\Delta^{2}\,\left(1-a^{2}\right)\,\left(3-a\right)\right]},$$

$$p^{M-S^{*}}=\frac{\,\left[6\,C_{L}-\beta \,\Delta^{2}\,\left(1-a^{2}\right)\,\left(3-a\right)\right]\,\left[\alpha_{0}+t\,\left(f-\alpha_{0}\right)\right]\,\alpha_{0}+2\,C_{L}\,\beta \,c_{m}}{\beta \,\left[8\,C_{L}-\beta \,\Delta^{2}\,\left(1-a^{2}\right)\,\left(3-a\right)\right]},$$

$\tau_{m}^{M-S^{*}}=\frac{\Delta \,\left(1-a^{2}\right)\,\left[\alpha_{0}+t\,\left(f-\alpha_{0}\right)-\beta \,c_{m}\right]}{8\,C_{L}-\beta \,\Delta^{2}\,\left(1-a^{2}\right)\,\left(3-a\right)}$ and $\tau_{r}^{M-S^{*}}=\frac{\Delta \,\left(1-a^{2}\right)\,\left[\alpha_{0}+t\,\left(f-\alpha_{0}\right)-\beta \,c_{m}\right]}{8\,C_{L}-\beta \,\Delta^{2}\,\left(1-a^{2}\right)\,\left(3-a\right)}$

### Model R

Nowadays, there are more and more large retailers, such as the famous retailers (i.e., Wal-Mart, Carrefour, Su-Ning, and JD.com), gradually becoming the dominant force in the market. The following study structures a Stackelberg CLSC model with the retailer as the leader. Retailers are the closest members to consumers in CLSC. They always play an increasingly significant role in improving supply chain performance. Under this model, the retailer dominates the market and decides first in the game process. The manufacturer is the follower and makes decisions according to the decisions of the retailer. The retailer and manufacturer are still competing in the reverse channel for recycling used products. Then the manufacturer takes on the remanufacturing business.

At this point, we set the game sequence of the model as follows:

(1) The retailer first determines the retail price *p* of new and remanufactured products and the retailer’s recycling rate τ_*r*_ for used products.
(2) Then, the manufacturer determines the wholesale price *w* of new and remanufactured products, the manufacturer’s recycling rate τ_*m*_ for used products and the transfer fee *b* to the retailer.

**Propositions 3:** When the retailer is the market leader without information sharing, the optimal decisions of the manufacturer and retailer are as follows:


$$w^{R-N^{*}}=\frac{\left[2\,C_{L}-\beta \,\Delta^{2}\,\left(1-a^{2}\right)\right]\,\alpha_{0}+\left[6\,C_{L}-\beta \,\Delta^{2}\,\left(1-a^{2}\right)\,\left(1-a\right)\right]\,\beta \,c_{m}}{\beta \,\left[8\,C_{L}-\beta \,\Delta^{2}\,\left(1-a^{2}\right)\,\left(2-a\right)\right]},$$

$$p^{R-N^{*}}=\frac{\left[6\,C_{L}-\beta \,\Delta^{2}\,\left(1-a^{2}\right)\,\left(2-a\right)\right]\,\left[\alpha_{0}+t\,\left(f-\alpha_{0}\right)\right]+2\,C_{L}\,\beta \,c_{m}}{\beta \,\left[8\,C_{L}-\beta \,\Delta^{2}\,\left(1-a^{2}\right)\,\left(2-a\right)\right]},$$

$\tau_{m}^{R-N^{*}}=\frac{\Delta \,\left(1-a^{2}\right)\,\left(\alpha_{0}-\beta \,c_{m}\right)}{8\,C_{L}-\beta \,\Delta^{2}\,\left(1-a^{2}\right)\,\left(2-a\right)}$ and $\tau_{r}^{R-N^{*}}=\frac{\Delta \,\left(1-a^{2}\right)\,\left(\alpha_{0}-\beta \,c_{m}\right)}{8\,C_{L}-\beta \,\Delta^{2}\,\left(1-a^{2}\right)\,\left(2-a\right)}+$$\frac{\Delta \,\left(1-a^{2}\right)\,t\,\left(f-\alpha_{0}\right)}{8\,C_{L}-\beta \,\Delta^{2}\,\left(1-a^{2}\right)\,\left(2-a\right)}$

**Propositions 4:** When the retailer is the market leader with information sharing, the optimal decisions of the manufacturer and retailer are as follows:


$$w^{R-S^{*}}=\frac{\,\left[2\,C_{L}-\beta \,\Delta^{2}\,\left(1-a^{2}\right)\right]\,\left[\alpha_{0}+t\,\left(f-\alpha_{0}\right)\right]+\left[6\,C_{L}-\beta \,\Delta^{2}\,\left(1-a^{2}\right)\,\left(1-a\right)\right]\,\beta \,c_{m}}{\beta \,\left[8\,C_{L}-\beta \,\Delta^{2}\,\left(1-a^{2}\right)\,\left(2-a\right)\right]},$$

$$p^{R-S^{*}}=\frac{\,\left[6\,C_{L}-\beta \,\Delta^{2}\,\left(1-a^{2}\right)\,\left(2-a\right)\right]\,\left[\alpha_{0}+t\,\left(f-\alpha_{0}\right)\right]+2\,C_{L}\,\beta \,c_{m}}{\beta \,\left[8\,C_{L}-\beta \,\Delta^{2}\,\left(1-a^{2}\right)\,\left(2-a\right)\right]},$$

$\tau_{m}^{R-S^{*}}=\frac{\Delta \,\left(1-a^{2}\right)\,\left[\alpha_{0}+t\,\left(f-\alpha_{0}\right)-\beta \,c_{m}\right]}{8\,C_{L}-\beta \,\Delta^{2}\,\left(1-a^{2}\right)\,\left(2-a\right)}$ and $\tau_{r}^{R-S^{*}}=\frac{\Delta \,\left(1-a^{2}\right)\,\left[\alpha_{0}+t\,\left(f-\alpha_{0}\right)-\beta \,c_{m}\right]}{8\,C_{L}-\beta \,\Delta^{2}\,\left(1-a^{2}\right)\,\left(2-a\right)}$

### Model M–R

In reality, although market forces gradually shift to retailers, retailers are not always strong enough to dominate the market. There are also situations where manufacturers and retailers are evenly matched in the market, where they make decisions at the same time. In this mode, the manufacturer and the retailer still conduct competitive recycling of used products in the market. The manufacturer undertakes the business of recycling used products for reproduction. Apple, for example, and its retailers recycle used phones and computers at the same time.

At this point, we set the game sequence of the model as follows:

The manufacturer determines the wholesale price *w*, the recycling rate τ_*m*_ and the transfer price *b* to the retailer. The retailer simultaneously determines the retail price *p* and the recycling rate τ_*r*_.

**Propositions 5:** The optimal decisions of the manufacturer and retailer are as follows in the Nash game between the manufacturer and the retailer without information sharing:


$$w^{M-R-N^{*}}=\frac{\left[2\,C_{L}-\beta \,\Delta^{2}\,\left(1-a^{2}\right)\right]\,\alpha_{0}+\left[4\,C_{L}-\beta \,\Delta^{2}\,\left(1-a^{2}\right)\right]\,\beta \,c_{m}}{\beta \,\left[6\,C_{L}-2\,\beta \,\Delta^{2}\,\left(1-a^{2}\right)\right]},$$

$$p^{M-R-N^{*}}=\frac{\left[4\,C_{L}-2\,\beta \,\Delta^{2}\,\left(1-a^{2}\right)\right]\,\left[\alpha_{0}+t\,\left(f-\alpha_{0}\right)\right]+2\,C_{L}\,\beta \,c_{m}}{\beta \,\left[6\,C_{L}-2\,\beta \,\Delta^{2}\,\left(1-a^{2}\right)\right]},$$

$\tau_{m}^{M-R-N^{*}}=\frac{\Delta \,\left(1-a^{2}\right)\,\left(\alpha_{0}-\beta \,c_{m}\right)}{6\,C_{L}-2\,\beta \,\Delta^{2}\,\left(1-a^{2}\right)}$ and $\tau_{r}^{M-R-N^{*}}=\frac{\Delta \,\left(1-a^{2}\right)\,\left[\alpha_{0}+t\,\left(f-\alpha_{0}\right)-\beta \,c_{m}\right]}{6\,C_{L}-2\,\beta \,\Delta^{2}\,\left(1-a^{2}\right)}$

**Propositions 6:** The optimal decisions of the manufacturer and retailer are as follows in the Nash game between the manufacturer and the retailer with information sharing:


$$w^{M-R-S^{*}}=\frac{\,\left[2\,C_{L}-\beta \,\Delta^{2}\,\left(1-a^{2}\right)\right]\,\left[\alpha_{0}+t\,\left(f-\alpha_{0}\right)\right]+\left[4\,C_{L}-\beta \,\Delta^{2}\,\left(1-a^{2}\right)\right]\,\beta \,c_{m}}{\beta \,\left[6\,C_{L}-2\,\beta \,\Delta^{2}\,\left(1-a^{2}\right)\right]},$$

$$p^{M-R-S^{*}}=\frac{\left[4\,C_{L}-2\,\beta \,\Delta^{2}\,\left(1-a^{2}\right)\right]\,\left[\alpha_{0}+t\,\left(f-\alpha_{0}\right)\right]+2\,C_{L}\,\beta \,c_{m}}{\beta \,\left[6\,C_{L}-2\,\beta \,\Delta^{2}\,\left(1-a^{2}\right)\right]},$$

$\tau_{m}^{M-R-S^{*}}=\frac{\Delta \,\left(1-a^{2}\right)\,\left[\alpha_{0}+t\,\left(f-\alpha_{0}\right)-\beta \,c_{m}\right]}{6\,C_{L}-2\,\beta \,\Delta^{2}\,\left(1-a^{2}\right)}$ and $\tau_{r}^{M-R-S^{*}}=\frac{\Delta \,\left(1-a^{2}\right)\,\left[\alpha_{0}+t\,\left(f-\alpha_{0}\right)-\beta \,c_{m}\right]}{6\,C_{L}-2\,\beta \,\Delta^{2}\,\left(1-a^{2}\right)}$

In this section, we obtain optimal decisions of the manufacturer and retailer using the backward induction method. We present these optimal decisions of the manufacturer and retailer and their profits in Supplementary Appendix Tables 1, 2. We then compare these results and present some interesting findings.

## Comparison and Analysis of Results

In this section, we compare the results in Model M, Model R, and Model M–R. Moreover, we analyzed these results under the three power structures with or without information sharing and got the following propositions.

**Proposition 7:** The relationship of the optimal wholesale price is *w*^*M*^ > *w*^*M*−*N*^ > *w*^*R*^. When *f* > α_0_, *w*^*S*^ > *w*^*N*^, on the contrary *w*^*S*^ ≤ *w*^*N*^ when *f* ≤ α_0_.

The manufacturer’s profit is determined by the difference between the production cost and the wholesale price. When production cost is fixed, a higher wholesale price meets the manufacturer’s expectation. Accordingly, when the manufacturer plays a leading role, the wholesale price is the highest. In the market, where the retailer dominates the market, the retailer takes priority in decision-making. Because the retailer is closer to the consumer, she can use its structural advantages and market forces to lower the wholesale price to improve her profit. On the contrary, when the manufacturer is the market leader, he can use his market power to set higher wholesale prices for higher profits.

Moreover, market demand affects the wholesale price from the manufacturer’s point of view. In the game between the manufacturer and consumers, when the market demand is low, the manufacturer reduces the wholesale price to reduce the possibility of unmarketable products. Instead, when demand is high, the manufacturer raises wholesale prices because he does not need to worry about sales. If the manufacturer finds that the demand that the retailer shares with him is higher than he knows, the manufacturer raises wholesale prices. At this point, the manufacturer knows that raising wholesale prices has only a small effect on profits because demand is enough. In contrast, if the manufacturer finds that the market demand is lower than he knows, he cuts the wholesale price for the sake of stimulating retailer to wholesale more products to not generate an inventory.

**Proposition 8:** The relationship of the optimal retail price is *p*^*R*^ > *p*^*M*^ > *p*^*M*−*N*^, and *p*^*S*^ = *p*^*N*^.

The retail price improves with the accuracy of retailers’ forecasts of market demand. When the retailer is the market leader, she sets higher retail price to increase her profits. At this point, the difference in retail prices is not significant whether the manufacturer dominates the market or the retailer dominates the market. However, the retailer gets a higher profit when selling the same unit of products since the retailer can use her market power to force the manufacturer to set a lower wholesale price. The retailer sets the best retail price according to demand information she predicts when setting the wholesale price. Therefore, information-sharing decisions do not affect retail price, but it affects manufacturers’ decisions about the wholesale price.

The retailer’s information-sharing decision depends on the demand information she predicts. The retailer chooses not to share her forecast market information because it increases the wholesale price when the predicted market demand is large. When market demand observed by the retailer is small, the manufacturer reduces the wholesale price to simulate the retailer to sell products for the sake of avoiding loss caused by the small market demand. At this time, the retailer gains a higher unit profit, so the retailer decides to share her forecast market demand. The retailer formulates strategies to reduce the wholesale price for the sake of obtaining higher profits at a given wholesale price. We can get Corollary 1.

**Corollary 1:** Under a particular power structure, the retailer shares her forecast demand information if the market demand is low.

The retailer has the power to make an information-sharing decision. She makes decisions that tend to maximize her profits. The retailer chooses not to share her forecast demand information when her forecast market demand is large. However, she decides to share her forecast demand information when her forecast market demand is low. This is because that the wholesale price is directly proportional to the perceived market demand. The wholesale price is higher when the perceived market demand is higher. Higher wholesale price cuts into retailer’s profits. Therefore, the retailer conceals her forecast demand information from the manufacturer for the sake of preventing the manufacturer from raising the wholesale price when the demand predicted by the retailer is higher than the demand known by the manufacturer.

**Proposition 9:** Under three power structures, the relationship between the manufacturer’s and retailer’s recycling rates is, respectively, $\tau_{m}^{R}<\tau_{m}^{M}<\tau_{m}^{M-R}$ and $\tau_{r}^{R}<\tau_{r}^{M}<\tau_{r}^{M-R}$. The retailer’s information-sharing decision influences the manufacturer’s recovery rate, which is $\tau_{m}^{N}<\tau_{m}^{S}$ if *f* > α_0_, otherwise,$\tau_{m}^{N}\geq \tau_{m}^{S}$ if *f* ≤ α_0_. The retailer’s information-sharing decision does not influence retailer’s recycling rate, i.e., $\tau_{r}^{N}=\tau {}_{r}^{S}$.

From proposition 9, we know that the manufacturer and retailer have the highest recycling rate in the Nash game model. From the supply chain perspective, the Nash game has an optimal recycling rate. The manufacturer and retailer have the lowest recycling rate for used products when the retailer dominates the market. From the perspective of information sharing, information sharing has no influence on the retailer’s recycling rate for used products. While information sharing affects the manufacturer’s rate.

Comprehensive information sharing affects the manufacturer’s decisions. Information sharing itself is a part of the retailer’s decision, and the retailer must have taken information sharing into consideration when making other decisions. Therefore, from the result, it is concluded that information sharing has no impact on the retailer’s decision. Nevertheless, information sharing still affects the profits of the manufacturer and retailer because information sharing affects the manufacturer’s decisions.

**Proposition 10:** When the retailer shares her forecast demand information, $\tau_{r}^{S}=\tau_{m}^{S}$. When the retailer chooses not to share her forecast demand information, if *f* > α_0_, $\tau_{r}^{S}>\tau_{m}^{S}$; Otherwise, $\tau_{r}^{S}\leq \tau {}_{m}^{S}$.

The recycling rates of the manufacturer and retailer are the same and increased with the accuracy of the forecast when the retailer shares her forecast demand information. When the retailer chooses not to share her forecast demand information, if the retailer observes a large market demand, the retailer’s recycling rate is higher than that of the manufacturer. Conversely, the manufacturer’s recycling rate is higher than the retailer’s if the retailer observes a smaller market demand, which is consistent with Corollary 1.

The accuracy of forecasting market demand affects supply chain participants expected profit level. The manufacturer and retailer gain higher profit margins as forecasting accuracy improves. Therefore, the way to enhance the performance of the supply chain is to improve the accuracy of retailers’ prediction of uncertain market demand.

**Proposition 11:** The relationship of the manufacturer’s profit is $\pi_{m}^{M}>\pi_{m}^{M-R}>\pi_{m}^{R}$; The relationship of the retailer’s profit is $\pi_{r}^{R}>\pi_{r}^{M-R}>\pi {}_{r}^{M}$.

Market demand determines the retail price, while the manufacturer’s aware demand determines wholesale price according to Propositions 7 and 8. In terms of the channel power structure, the manufacturer’s profit is the largest when the manufacturer dominates the market; analogously, the retailer’s profit is the largest when the retailer dominates the market. Because members with dominant market forces can use their power to make decisions that are most favorable to them. For example, when the retailer is the leader in the market, she can use her power to compel the manufacturer to reduce wholesale price while raising retail price. It can be seen that the market power structure has a great impact on the profit level of participants in CLSC. In general, leaders in the market can make more profits than those in other power structures.

**Proposition 12:** The relationship of market demand is *D*^*M*−*R*^ > *D*^*M*^ > *D*^*R*^.

According to economic theory, market demand is inversely proportional to the retail price. According to Proposition 8, the retail price of products is the lowest and the consumer surplus is the largest in the Nash game model. Therefore, demand is greatest in Model M–R. From the perspective of market demand, the maximum market demand is definitely the best choice for the supply chain.

The wholesale price and retail price of products, the recycling rate of used products, and the profit level of participants all increase with the improvement of the accuracy of retailers’ prediction of uncertain market demand. Therefore, the coordination of the supply chain always tends to balance the market power of the manufacturer and retailer.

We discuss how the retailer’s information-sharing decision affects other decisions and profits of the manufacturer and retailer in this section. According to the above analysis, we know that information-sharing decisions are not always inclined to happen in CLSC. We discuss when the retailer shares her forecast demand information in the next section.

## The Value of Information Sharing

We discuss when the retailer shares her forecast demand information in this section. According to the last section, the retailer’s information-sharing decision is related to her profits. She has an incentive to share information if it makes her more profitable, or at least not less profitable. Next, we present the effect of information sharing on profit of supply chain under three power structures.

The information value to the manufacturer when the retailer shares forecast demand information in Model M, Model R, and Model M–R:


$$V_{m}^{M}=\pi_{m}^{M-S}-\pi_{m}^{M-N}=\frac{C_{L}\,k\,t\,\left[8\,C_{L}-\beta \,\Delta^{2}\,\left(1-a^{2}\right)\,\left(3-2\,a\right)\right]}{\beta \,{\left[8\,C_{L}-\beta \,\Delta^{2}\,\left(1-a^{2}\right)\,\left(3-a\right)\right]}^{2}},$$

$$V_{m}^{R}=\pi_{m}^{R-S}-\pi_{m}^{R-N}=\frac{C_{L}\,k\,t\,\left[4\,C_{L}-\beta \,\Delta^{2}\,\left(1-a^{2}\right)\right]}{\beta \,{\left[8\,C_{L}-\beta \,\Delta^{2}\,\left(1-a^{2}\right)\,\left(2-a\right)\right]}^{2}},$$

$$V_{m}^{M-R}=\pi_{m}^{M-R-S}-\pi_{m}^{M-R-N}=\frac{C_{L}\,k\,t\,\left[4\,C_{L}-\beta \,\Delta^{2}\,\left(1-a^{2}\right)\right]}{\beta \,{\left[6\,C_{L}-2\,\beta \,\Delta^{2}\,\left(1-a^{2}\right)\right]}^{2}}$$

**Proposition 13:** Information sharing enhances the manufacture’s profits under three channel power structures.

Under the certain channel power structure, the retailer shares forecast market demand information, which increases manufacturer’s profit. The increase in manufacturer’s profits is in direct proportion to the accuracy of retailer’s forecasts of market demand.

The information value to the retailer when the retailer shares forecast market demand information in Model M, Model R, and Model M–R:


$$V_{r}^{M}=\pi_{r}^{M-S}-\pi_{r}^{M-N}=\frac{-C_{L}\,k\,t\,\left[8\,C_{L}-\beta \,\Delta^{2}\,\left(1-a^{2}\right)\,\left(3-3\,a\right)\right]}{\beta \,{\left[8\,C_{L}-\beta \,\Delta^{2}\,\left(1-a^{2}\right)\,\left(3-a\right)\right]}^{2}},$$

$$V_{r}^{R}=\pi_{r}^{R-S}-\pi_{r}^{R-N}=\frac{-C_{L}\,k\,t\,\left[4\,C_{L}-\beta \,\Delta^{2}\,\left(1-a^{2}\right)\,\left(2-a\right)\right]}{\beta \,{\left[8\,C_{L}-\beta \,\Delta^{2}\,\left(1-a^{2}\right)\,\left(2-a\right)\right]}^{2}},$$

$$V_{r}^{M-R}=\pi_{r}^{M-R-S}-\pi_{r}^{M-R-N}=$$

$$\frac{-C_{L}\,k\,t\,\left[4\,C_{L}-\beta \,\Delta^{2}\,\left(1-a^{2}\right)\,\left(2-a\right)\right]}{\beta \,{\left[6\,C_{L}-2\,\beta \,\Delta^{2}\,\left(1-a^{2}\right)\right]}^{2}}$$

**Proposition 14:** Information sharing reduces the retailer’s profits under three channel power structure.

The retailer’s choice to share forecast demand information with manufacturers reduces the retailer’s profit. But when the retailer is the leader in the market, the reduction in retailer’s profits is the least.

Since the retailer shares the forecast market demand information, the retailer’s profit gets lower. Therefore, without any compensation, retailer chooses not to share forecast market demand information. The manufacturer must pay the retailer some compensation to offset the retailer’s profit loss for the sake of encouraging retailer to share her forecasts demand information. However, the compensation that the manufacturer pays to retailer should not be higher than the value of the information added by the forecast information shared by the retailer. Therefore, retailer is likely to share forecast market demand if information sharing increases the profit of the supply chain system.

The value to the supply chain system when the retailer shares forecast market demand information in Model M, Model R, and Model M–R:


$$V_{s}^{M}=V_{m}^{M}+V_{r}^{M}=\frac{-C_{L}\,k\,t\,a\,\Delta^{2}\,\left(1-a^{2}\right)}{{\left[8\,C_{L}-\beta \,\Delta^{2}\,\left(1-a^{2}\right)\,\left(3-a\right)\right]}^{2}}<0,$$

$$V_{s}^{R}=V_{m}^{R}+V_{r}^{R}=\frac{C_{L}\,k\,t\,\Delta^{2}\,\left(1-a^{2}\right)\,\left(1-a\right)}{{\left[8\,C_{L}-\beta \,\Delta^{2}\,\left(1-a^{2}\right)\,\left(2-a\right)\right]}^{2}}>0,$$

$$V_{s}^{M-R}=V_{m}^{M-R}+V_{r}^{M-R}=\frac{C_{L}\,k\,t\,\Delta^{2}\,\left(1-a^{2}\right)\,\left(1-a\right)}{{\left[6\,C_{L}-2\,\beta \,\Delta^{2}\,\left(1-a^{2}\right)\right]}^{2}}>0$$

**Proposition 15:** Information sharing enhances the profit of supply chain when the manufacture does not play leading role.

Information sharing reduces the overall profit of the supply chain when the manufacturer dominates the market. Therefore, in this condition, the retailer chooses not to share forecast demand information is the optimal decision for CLSC. Since manufacturer’s dominant position in the market reduces the profit of the retailer, the profit level is lower if retailer shares forecast demand information. Information sharing is beneficial to the manufacturer, but is bad for retailer. From the manufacturer’s point of view, if the manufacturer pays a certain amount of compensation to the retailer to offset the retailer’s profit loss, the manufacturer’s profit is less than if he does not accept the information sharing, so the manufacturer chooses not to accept information sharing. From the perspective of the retailer, if the manufacturer pays the retailer compensation without reducing his profit, then the maximum compensation that the retailer can obtain is not enough to make up for the decrease of the profit. Therefore, the retailer refuses to share proprietary information about their forecasts. From the perspective of supply chain performance, when the manufacturer dominates the market, the retailer shares the forecast market demand, which reduces the profit of the supply chain.

When the manufacturer does not play a leading role, information sharing improves the supply chain’s profit. At this point, retailer has an incentive to share the forecast market demand information because the manufacturer has the ability to offset the loss of the retailer caused by information sharing.

**Corollary 2:** If the manufacturer does not play a leading role, the retailer shares her forecast market demand information when the manufacturer gives her a certain subsidy.

Through Propositions 13, 14, and 15, we can find that the retailer shares forecast market demand information if the retailer dominates the market or the manufacturer and retailer are evenly matched in the market. The retailer and manufacturer conduct competitive recycling of used products at the same time in CLSC with dual recycling channels, retailer decides not to share forecast market demand information when the manufacturer dominates the market. When the retailer is a leader or the manufacture and retailer are evenly matched in the market, because information sharing improves supply chain’s profit, retailer shares forecast demand information when the manufacturer gives her a certain subsidy.

The retailer’s first goal must be to protect her own interests. Based on the above analysis, we can draw the most important conclusion of this study. The retailer only shares forecast demand information when the manufacturer is not the leader and the market demand is relatively low.

## Numerical Examples

We present numerical examples to compare three channel power structures model with/without information sharing. Then we calculated *C*_*L*_ = 1000, β = 0.3, *c*_*m*_ = 20, *c*_*r*_ = 15, *b* = Δ = 5, *a* = 0.7, *f* = 150, α_0_ = 100, *k* = 10, based on Huang et al. (2013) assignment, which is used for all of the parameters in this study. We intuitively show the relationship among variables (information sharing, wholesale price, retail price, and profit and information value).

**FIGURE 2:**
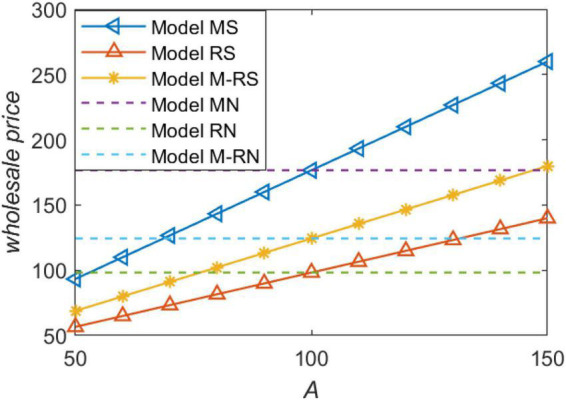
The wholesale price with forecast demand.

We show the relationship between the wholesale price and forecast demand in Figure 2. If we set the situation without information sharing as the benchmark model, we can know from Figure 1 that the wholesale price is higher than the benchmark model no matter under which channel power structure when the market demand predicted by the retailer is higher (i.e., *f* > α_0_). Consistent with Proposition 1, what is important to our conclusion is that, regardless of the channel power structure, wholesale price is lower than the benchmark model when the market demand predicted by the retailer is lower (i.e., *f* < α_0_). We must make it clear that lower wholesale price is definitely a better option for the retailer when the retailer only consider price. Therefore, the retailer chooses to hide the demand information to avoid the manufacturer raising the wholesale price when the market demand is high. The wholesale price is the highest when the manufacturer is the market leader.

**FIGURE 3:**
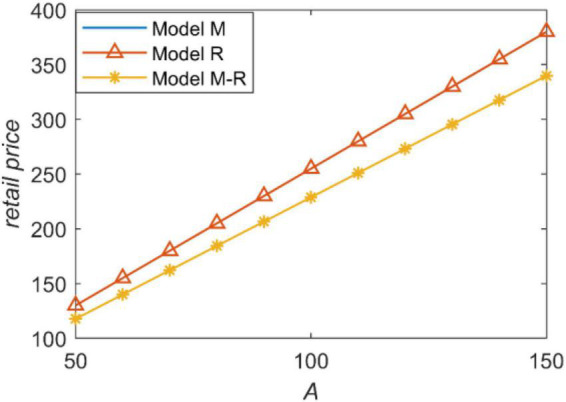
The retail price with forecast demand.

Figure 3 shows that the retailer price is higher with the increase of forecast market demand. This conclusion is consistent with the classical cognition in economics. Regardless of channel power structures, the information-sharing decision does not affect the retail price, because the retailer decides the information-sharing decision and the retail price at the same time. Therefore, the retailer hopes that the wholesale price is low.

**FIGURE 4:**
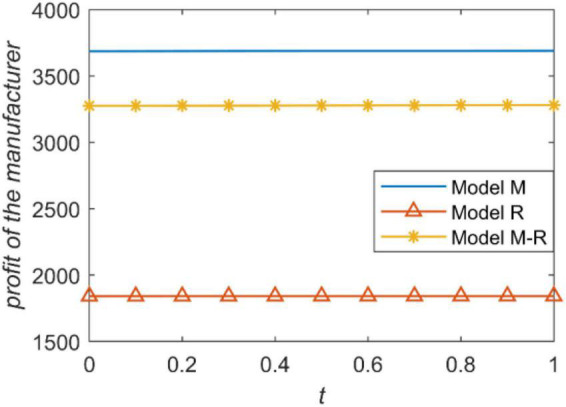
The manufacturer’s profit with prediction accuracy.

**FIGURE 5:**
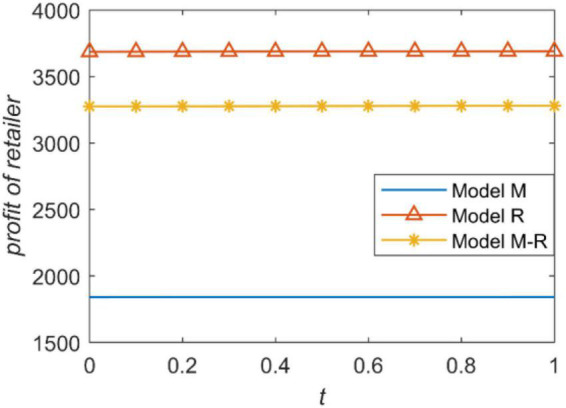
The retailer’s profit with prediction accuracy.

We present the relationship between the accuracy of forecast demand information and the profit of the manufacturer and retailer in Figures 4, 5. The profit of a supply chain member is proportional to the power he has. For example, when the manufacturer is the leader, he has the highest power and gets the highest profit. When the retailer is the leader, the manufacturer also gets the lowest profit because he has the least power. The retailer’s profit is the opposite. Figures 4, 5 intuitively reflect that channel power structure has a greater impact on the profits of the supply chain rather than the accuracy of information prediction.

**FIGURE 6:**
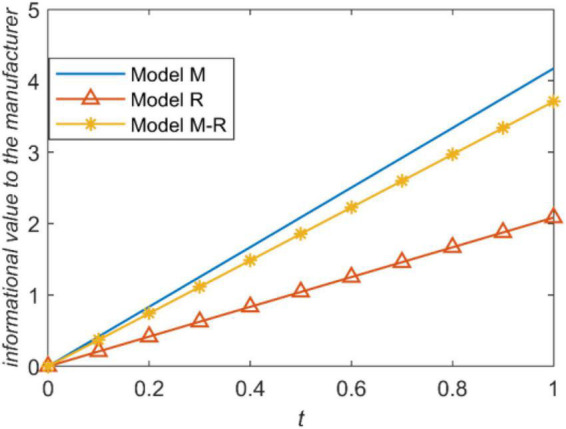
Influence of prediction accuracy on manufacturer’s profit.

We present the influence of prediction accuracy of demand information on the manufacture’s profit under three power structures, as shown in Figure 6. According to Figure 5, information sharing brings the most value to the manufacturer when the manufacturer is the leader; information sharing brings the least value to the manufacturer when the retailer is the leader. Consistent with Proposition 7, information sharing always enhance the manufacturer’s profit. The main reason that information sharing brings different information values to the manufacturer under different channel power structures is the influence of information sharing on wholesale price.

## Conclusion

We structure a game model to compare and analyze the optimal decision under three channel power structures. We build a CLSC model that only has a manufacturer and a retailer. The retailer can predict market demand information and decide whether to share forecast demand information. This study analyses the impact of dual recycling channels on optimal information-sharing decisions in CLSC under three power structures.

We explicitly characterize the role of information sharing in a CLSC. Surprisingly, we find that when the manufacturer does not play a leading role, the retailer shares her forecast demand information with the manufacturer if the market demand is low. We also show that information sharing reduces the overall profit of the supply chain when the manufacturer dominates the market. In addition, our results also illustrate that information sharing affects supply chain profits by affecting the wholesale price. We make a contribution to the information-sharing literature by integrating the existing information-sharing model with dual recycling channels and channel power structure. Finally, this study provides some valuable insights into the retailer’s information-sharing decision.

Information always is an important influencing factor for enterprises to make decisions. However, in reality, it is difficult for enterprises to obtain complete market information because when enterprises have private information, they can bring additional benefits, enterprises generally conceal their proprietary information from other participants. Motivating participants to share demand information is an important means to enhance supply chain performance. The conclusions are all based on the assumptions of this study, and some of the assumptions are only put forward to simplify the operation, which may not be consistent with the actual situation in the market. Future research can refine the model by relaxing the assumptions mentioned in this study. For example, Apple adopts a differential pricing strategy depending on the consumers’ willingness to pay for new and remanufactured products. Future research can study how the information-sharing decisions of retailers in differential pricing affect the optimal decisions in CLSC with dual-recycling channels. We only consider the retailer’s forecast for uncertain part of market demand. Future research can consider how the information-sharing decisions of manufacturers and retailers affect the supply chain coordination when they simultaneously forecast the uncertain part of market demand. In general, used products are heterogeneous. However, our research regards used products as homogeneity, and future research can consider heterogeneous used products.

## Data Availability Statement

The original contributions presented in this study are included in the article/Supplementary Material, further inquiries can be directed to the corresponding author/s.

## Author Contributions

HZ was responsible for conceptualization and methodology of the manuscript. XH was responsible for supervision, project administration, and validation of the manuscript. XC writing the original draft of the manuscript, and revised the manuscript according to the comments of reviewers. All authors contributed to the manuscript and approved the submitted.

## Conflict of Interest

The authors declare that the research was conducted in the absence of any commercial or financial relationships that could be construed as a potential conflict of interest.

## Publisher’s Note

All claims expressed in this article are solely those of the authors and do not necessarily represent those of their affiliated organizations, or those of the publisher, the editors and the reviewers. Any product that may be evaluated in this article, or claim that may be made by its manufacturer, is not guaranteed or endorsed by the publisher.
