# Supplementary material for: To Share or Not to Share? The Role of Retailer’s Information Sharing in a Closed-Loop Supply Chain
Source: Front Psychol. 2022 Jul 18;13:941952. doi: 10.3389/fpsyg.2022.941952 (PMC9341282; doi:10.3389/fpsyg.2022.941952)
Supplement: Supplementary file 1 [file Table_1.DOCX]

**Appendix A**

**Table A1.** The optimal solutions under three power structures without information sharing

| The optimal value | Model M | Model R | Model M-R |
| --- | --- | --- | --- |
| $w$ | $\frac{\left[4C_{L}-\beta\Delta^{2}\left(1-a^{2}\right)\left(2-a\right)\right]\alpha_{0}+\left[4C_{L}-\beta\Delta^{2}\left(1-a^{2}\right)\right]\beta c_{m}}{\beta\left[8C_{L}-\beta\Delta^{2}\left(1-a^{2}\right)\left(3-a\right)\right]}$ | $\frac{\left[2C_{L}-\beta\Delta^{2}\left(1-a^{2}\right)\right]\alpha_{0}+\left[6C_{L}-\beta\Delta^{2}\left(1-a^{2}\right)\left(1-a\right)\right]\beta c_{m}}{\beta\left[8C_{L}-\beta\Delta^{2}\left(1-a^{2}\right)\left(2-a\right)\right]}$ | $\frac{\left[2C_{L}-\beta\Delta^{2}\left(1-a^{2}\right)\right]\alpha_{0}+\left[4C_{L}-\beta\Delta^{2}\left(1-a^{2}\right)\right]\beta c_{m}}{\beta\left[6C_{L}-2\beta\Delta^{2}\left(1-a^{2}\right)\right]}$ |
| $p$ | $\frac{\left[6C_{L}-\beta\Delta^{2}\left(1-a^{2}\right)\left(3-a\right)\right]A+2C_{L}\beta c_{m}}{\beta\left[8C_{L}-\beta\Delta^{2}\left(1-a^{2}\right)\left(3-a\right)\right]}$ | $\frac{\left[6C_{L}-\beta\Delta^{2}\left(1-a^{2}\right)\left(2-a\right)\right]A+2C_{L}\beta c_{m}}{\beta\left[8C_{L}-\beta\Delta^{2}\left(1-a^{2}\right)\left(2-a\right)\right]}$ | $\frac{\left[4C_{L}-2\beta\Delta^{2}\left(1-a^{2}\right)\right]A+2C_{L}\beta c_{m}}{\beta\left[6C_{L}-2\beta\Delta^{2}\left(1-a^{2}\right)\right]}$ |
| $\tau_{m}$ | $\frac{\Delta\left(1-a^{2}\right)\left(\alpha_{0}-\beta c_{m}\right)}{8C_{L}-\beta\Delta^{2}\left(1-a^{2}\right)\left(3-a\right)}$ | $\frac{\Delta\left(1-a^{2}\right)\left(\alpha_{0}-\beta c_{m}\right)}{8C_{L}-\beta\Delta^{2}\left(1-a^{2}\right)\left(2-a\right)}$ | $\frac{\Delta\left(1-a^{2}\right)\left(\alpha_{0}-\beta c_{m}\right)}{6C_{L}-2\beta\Delta^{2}\left(1-a^{2}\right)}$ |
| $\tau_{r}$ | $\frac{\Delta\left(1-a^{2}\right)\left(A-\beta c_{m}\right)}{8C_{L}-\beta\Delta^{2}\left(1-a^{2}\right)\left(3-a\right)}$ | $\frac{\Delta\left(1-a^{2}\right)\left(A-\beta c_{m}\right)}{8C_{L}-\beta\Delta^{2}\left(1-a^{2}\right)\left(2-a\right)}$ | $\frac{\Delta\left(1-a^{2}\right)\left(A-\beta c_{m}\right)}{6C_{L}-2\beta\Delta^{2}\left(1-a^{2}\right)}$ |
| $\pi_{m}$ |  | $\frac{C_{L}\left(\alpha_{0}-\beta c_{m}\right)^{2}\left[4C_{L}-\beta\Delta^{2}\left(1-a^{2}\right)\left(1+a\right)\right]-C_{L}kt\beta a\Delta^{2}\left(1-a^{2}\right)}{\beta\left[8C_{L}-\beta\Delta^{2}\left(1-a^{2}\right)\left(2-a\right)\right]^{2}}$ | $\frac{C_{L}\left(\alpha_{0}-\beta c_{m}\right)^{2}\left[4C_{L}-\beta\Delta^{2}\left(1-a^{2}\right)\left(1+a\right)\right]-C_{L}kt\beta a\Delta^{2}\left(1-a^{2}\right)}{\beta\left[6C_{L}-2\beta\Delta^{2}\left(1-a^{2}\right)\right]^{2}}$ |
| $\pi_{r}$ | $\frac{C_{L}\left[4C_{L}-\beta\Delta^{2}\left(1-a^{2}\right)\left(2+a\right)\right]\left(\alpha_{0}-\beta c_{m}\right)^{2}}{\beta\left[8C_{L}-\beta\Delta^{2}\left(1-a^{2}\right)\left(3-a\right)\right]^{2}}+\frac{C_{L}kt\left[12C_{L}-\beta\Delta^{2}\left(1-a^{2}\right)\left(5-2a\right)\right]}{\beta\left[8C_{L}-\beta\Delta^{2}\left(1-a^{2}\right)\left(3-a\right)\right]^{2}}$ | $\frac{C_{L}\left[8C_{L}-\beta\Delta^{2}\left(1-a^{2}\right)\left(1-a\right)\right]\left(\alpha_{0}-\beta c_{m}\right)^{2}}{\beta\left[8C_{L}-\beta\Delta^{2}\left(1-a^{2}\right)\left(2-a\right)\right]^{2}}+\frac{C_{L}kt\left[12C_{L}-\beta\Delta^{2}\left(1-a^{2}\right)\left(3-2a\right)\right]}{\beta\left[8C_{L}-\beta\Delta^{2}\left(1-a^{2}\right)\left(2-a\right)\right]^{2}}$ | $\frac{C_{L}\left(\alpha_{0}-\beta c_{m}\right)^{2}\left[4C_{L}-\beta\Delta^{2}\left(1-a^{2}\right)\left(1+a\right)\right]+C_{L}kt\left[8C_{L}-3\beta\Delta^{2}\left(1-a^{2}\right)\right]}{\beta\left[6C_{L}-2\beta\Delta^{2}\left(1-a^{2}\right)\right]^{2}}$ |

Note:$A=\alpha_{0}+t\left(f-\alpha_{0}\right)$.

**Table A2.** The optimal solutions under three power structures with information sharing

| The optimal value | Model M | Model R | Model M-R |
| --- | --- | --- | --- |
| $w$ | $\frac{\left[4C_{L}-\beta\Delta^{2}\left(1-a^{2}\right)\left(2-a\right)\right]A+\left[4C_{L}-\beta\Delta^{2}\left(1-a^{2}\right)\right]\beta c_{m}}{\beta\left[8C_{L}-\beta\Delta^{2}\left(1-a^{2}\right)\left(3-a\right)\right]}$ | $\frac{\left[2C_{L}-\beta\Delta^{2}\left(1-a^{2}\right)\right]A+\left[6C_{L}-\beta\Delta^{2}\left(1-a^{2}\right)\left(1-a\right)\right]\beta c_{m}}{\beta\left[8C_{L}-\beta\Delta^{2}\left(1-a^{2}\right)\left(2-a\right)\right]}$ | $\frac{\left[2C_{L}-\beta\Delta^{2}\left(1-a^{2}\right)\right]A+\left[4C_{L}-\beta\Delta^{2}\left(1-a^{2}\right)\right]\beta c_{m}}{\beta\left[6C_{L}-2\beta\Delta^{2}\left(1-a^{2}\right)\right]}$ |
| $p$ | $\frac{\left[6C_{L}-\beta\Delta^{2}\left(1-a^{2}\right)\left(3-a\right)\right]A+2C_{L}\beta c_{m}}{\beta\left[8C_{L}-\beta\Delta^{2}\left(1-a^{2}\right)\left(3-a\right)\right]}$ | $\frac{\left[6C_{L}-\beta\Delta^{2}\left(1-a^{2}\right)\left(2-a\right)\right]A+2C_{L}\beta c_{m}}{\beta\left[8C_{L}-\beta\Delta^{2}\left(1-a^{2}\right)\left(2-a\right)\right]}$ | $\frac{\left[4C_{L}-2\beta\Delta^{2}\left(1-a^{2}\right)\right]A+2C_{L}\beta c_{m}}{\beta\left[6C_{L}-2\beta\Delta^{2}\left(1-a^{2}\right)\right]}$ |
| $\tau_{m}$ | $\frac{\Delta\left(1-a^{2}\right)\left(A-\beta c_{m}\right)}{8C_{L}-\beta\Delta^{2}\left(1-a^{2}\right)\left(3-a\right)}$ | $\frac{\Delta\left(1-a^{2}\right)\left(\alpha_{0}-\beta c_{m}\right)}{8C_{L}-\beta\Delta^{2}\left(1-a^{2}\right)\left(2-a\right)}$ | $\frac{\Delta\left(1-a^{2}\right)\left(A-\beta c_{m}\right)}{6C_{L}-2\beta\Delta^{2}\left(1-a^{2}\right)}$ |
| $\tau_{r}$ | $\frac{\Delta\left(1-a^{2}\right)\left(A-\beta c_{m}\right)}{8C_{L}-\beta\Delta^{2}\left(1-a^{2}\right)\left(3-a\right)}$ | $\frac{\Delta\left(1-a^{2}\right)\left(A-\beta c_{m}\right)}{8C_{L}-\beta\Delta^{2}\left(1-a^{2}\right)\left(2-a\right)}$ | $\frac{\Delta\left(1-a^{2}\right)\left(A-\beta c_{m}\right)}{6C_{L}-2\beta\Delta^{2}\left(1-a^{2}\right)}$ |
| $\pi_{m}$ | $\frac{C_{L}\left(\alpha_{0}-\beta c_{m}\right)^{2}\text{+}C_{L}kt}{\beta\left[8C_{L}-\beta\Delta^{2}\left(1-a^{2}\right)\left(3-a\right)\right]}$ | $\frac{C_{L}\left[4C_{L}-\beta\Delta^{2}\left(1-a^{2}\right)\left(1+a\right)\right]\left[\left(\alpha_{0}-\beta c_{m}\right)^{2}+kt\right]}{\beta\left[8C_{L}-\beta\Delta^{2}\left(1-a^{2}\right)\left(2-a\right)\right]^{2}}$ | $\frac{C_{L}\left[4C_{L}-\beta\Delta^{2}\left(1-a^{2}\right)\left(1+a\right)\right]\left[\left(\alpha_{0}-\beta c_{m}\right)^{2}+kt\right]}{\beta\left[6C_{L}-2\beta\Delta^{2}\left(1-a^{2}\right)\right]^{2}}$ |
| $\pi_{r}$ | $\frac{C_{L}\left[4C_{L}-\beta\Delta^{2}\left(1-a^{2}\right)\left(2+a\right)\right]\left[\left(\alpha_{0}-\beta c_{m}\right)^{2}\text{+}kt\right]}{\beta\left[8C_{L}-\beta\Delta^{2}\left(1-a^{2}\right)\left(3-a\right)\right]^{2}}$ | $\frac{C_{L}\left[8C_{L}-\beta\Delta^{2}\left(1-a^{2}\right)\left(1-a\right)\right]\left[\left(\alpha_{0}-\beta c_{m}\right)^{2}\text{+}kt\right]}{\beta\left[8C_{L}-\beta\Delta^{2}\left(1-a^{2}\right)\left(2-a\right)\right]^{2}}$ | $\frac{C_{L}\left[4C_{L}-\beta\Delta^{2}\left(1-a^{2}\right)\left(1+a\right)\right]\left[\left(\alpha_{0}-\beta c_{m}\right)^{2}+kt\right]}{\beta\left[6C_{L}-2\beta\Delta^{2}\left(1-a^{2}\right)\right]^{2}}$ |

Note:$A=\alpha_{0}+t\left(f-\alpha_{0}\right)$.

**Appendix B**

**Proof of Model-M**

The Hessian matrix of $\pi_{m}$ is $H=\left(\begin{matrix} \frac{\partial\pi_{m}^{2}}{\partial\tau_{m}^{2}} & \frac{\partial\pi_{m}^{2}}{\partial\tau_{m}\partial w} \\ \frac{\partial\pi_{m}^{2}}{\partial\tau_{m}\partial w} & \frac{\partial\pi_{m}^{2}}{\partial w^{2}} \end{matrix}\right)=\left(\begin{matrix} \frac{-2C_{L}}{1-a^{2}} & -\beta\Delta\\ -\beta\Delta& -2\beta\end{matrix}\right)$.

For $\pi_{m}$ to be concave in $w$ and $\tau_{m}$, $\pi_{m}$ should satisfy conditions (i) $\partial\pi_{m}^{2}/\partial\tau_{m}^{2}<0$, (ii) $\partial\pi_{m}^{2}/\partial w^{2}<0$, (iii) $(\partial\pi_{m}^{2}/\partial\tau_{m}^{2})(\partial\pi_{m}^{2}/\partial w^{2})>(\partial\pi_{m}^{2}/\partial\tau_{m}\partial w)^{2}$. Note that (i) holds since $\partial\pi_{m}^{2}/\partial\tau_{m}^{2}\text{=}-2C_{L}/(1-a^{2})<0$ and $C_{L}>0$. Similarly, (ii) holds since $\partial\pi_{m}^{2}/\partial w^{2}\text{=}-2\beta<0$ and $\beta>0$. We need $(-2\beta)(-2C_{L}/(1-a^{2}))>\beta^{2}\Delta^{2}\Rightarrow4C_{L}>\beta\Delta^{2}(1-a^{2})$for condition (iii) to hold, which ambitions from the previous condition. Hence, $\pi_{m}$ is jointly concave in $\tau_{m}$ and $w$ if $4C_{L}>\beta\Delta^{2}(1-a^{2})$.

First, we prove that the manufacturer’s profit function of is jointly concave on $w$ and $\tau_{m}$ for a given $b$, which enables us to optimize w and m for the manufacturer of a given $b$, and then check the effect of $b$.

The Hessian matrix of $\pi_{r}$ is $H=\left(\begin{matrix} \frac{\partial\pi_{r}^{2}}{\partial\tau_{r}^{2}} & \frac{\partial\pi_{r}^{2}}{\partial\tau_{r}\partial p} \\ \frac{\partial\pi_{r}^{2}}{\partial\tau_{r}\partial p} & \frac{\partial\pi_{r}^{2}}{\partial p^{2}} \end{matrix}\right)=\left(\begin{matrix} \frac{-2C_{L}}{1-a^{2}} & -\beta b \\ -\beta b & -2\beta\end{matrix}\right)$.

For $\pi_{r}$ to be concave in $p$ and $\tau_{r}$, $\pi_{r}$ should satisfy conditions (i) $\partial\pi_{r}^{2}/\partial\tau_{r}^{2}<0$, (ii) $\partial\pi_{r}^{2}/\partial p^{2}<0$, (iii) $(\partial\pi_{r}^{2}/\partial\tau_{r}^{2})(\partial\pi_{r}^{2}/\partial p^{2})>(\partial\pi_{r}^{2}/\partial\tau_{r}\partial p)^{2}$. Note that (i) holds since $\partial\pi_{r}^{2}/\partial\tau_{r}^{2}\text{=}-2C_{L}/(1-a^{2})<0$ and $C_{L}>0$. Similarly, (ii) holds since $\partial\pi_{r}^{2}/\partial p^{2}\text{=-2}\beta<0$ and $\beta>0$. We need $(-2\beta)(-2C_{L}/(1-a^{2}))>\beta^{2}b^{2}\Rightarrow4C_{L}>\beta b^{2}(1-a^{2})$ for condition (iii) to hold, which ambitions from the previous condition. Hence, $\pi_{r}$ is jointly concave in $p$ and $\tau_{r}$ if $4C_{L}>\beta b^{2}(1-a^{2})$.

In the model-M, the manufacturer makes decisions in the game process in priority according to the retailer's reaction function. The retailer makes her decisions according to the decisions of the manufacturer. When selling products, retailer can only determine the retail price of products according to the wholesale price determined by the manufacturer. The manufacturer and retailer recycle used products at the same time. The manufacturer reproduces the used products, and then put the remanufactured products and new products on the market for sale.

We adopt the backward induction method. We first consider retailer's solution to the following problem for a given $w$, $\tau_{m}$ and $b$:

$\mathop{Max}\limits_{p,\tau_{r}} E(\pi_{r}\left|f\right.)=((p-w)(\alpha-\beta p)+b\tau_{r}(\alpha-\beta p)-\frac{C_{L}(\tau_{r}^{2}+a\tau_{m}^{2})}{1-a^{2}}\left|f\right.)$. (B1)

The retailer's price and recycling ratio are determined as follow:

$p^{M}=\frac{[\alpha_{0}+t(f-\alpha_{0})+\beta w]}{2\beta}-\frac{b^{2}(1-a^{2})[\alpha_{0}+t(f-\alpha_{0})-\beta w]}{2[4C_{L}-\beta b^{2}(1-a^{2})]}$,

$\tau_{r}^{M}=\frac{b(1-a^{2})[\alpha_{0}+t(f-\alpha_{0})-\beta w]}{[4C_{L}-\beta b^{2}(1-a^{2})]}$. (B2)

The model can be solved in two cases: Model-M without information sharing and Model-M with information sharing.

**Model-M without information sharing**

When retailer chooses to keep their forecasts of uncertain market demand secret from the manufacturer, manufacturer's expectations for retailer's price and recycling rate are as follow:

$E(p^{M-N})=\frac{(\alpha_{0}+\beta w)}{2\beta}-\frac{b^{2}(1-a^{2})(\alpha_{0}-\beta w)}{2[4C_{L}-\beta b^{2}(1-a^{2})]}$, $E(\tau_{r}^{M-N})=\frac{b(1-a^{2})(\alpha_{0}-\beta w)}{[4C_{L}-\beta b^{2}(1-a^{2})]}$. (B3)

The manufacturer's expected profit decision model is as follows:

$\mathop{Max}\limits_{w,\tau_{m},b} E(\pi_{m})=[w-c_{m}+\Delta(\tau_{m}+\tau_{r})](\alpha-\beta p)-\frac{C_{L}(\tau_{m}^{2}+a\tau_{r}^{2})}{1-a^{2}}-b\tau_{r}(\alpha-\beta p)$. (B4)

Then the manufacturer’s wholesale price and the recycling rate can be expressed as:

$w^{M-N}=\frac{[4C_{L}-\beta(\Delta^{2}+2\Delta b-ab^{2}-b^{2})(1-a^{2})]\alpha_{0}+[4C_{L}-\beta b^{2}(1-a^{2})]\beta c_{m}}{\beta[8C_{L}-\beta(\Delta^{2}+2\Delta b-ab^{2})(1-a^{2})]}$,

$\tau_{m}^{M-N}=\frac{\Delta(1-a^{2})(\alpha_{0}-\beta c_{m})}{[8C_{L}-\beta(\Delta^{2}+2\Delta b-ab^{2})(1-a^{2})]}$. (B5)

By substituting (B5) into (B3) and (B4) we can get the optimal solution $b=\frac{\Delta}{a}$. Since $0\leq a\leq1$ and $0\leq b\leq\Delta$, we make $b=\Delta$ and plug it in B5 to get the following results:

$w^{M-N^{*}}=\frac{[4C_{L}-\beta\Delta^{2}(1-a^{2})(2-a)]\alpha_{0}+[4C_{L}-\beta\Delta^{2}(1-a^{2})]\beta c_{m}}{\beta[8C_{L}-\beta\Delta^{2}(1-a^{2})(3-a)]}$,

$\tau_{m}^{M-N^{*}}=\frac{\Delta(1-a^{2})(\alpha_{0}-\beta c_{m})}{8C_{L}-\beta\Delta^{2}(1-a^{2})(3-a)}$. (B6)

Accordingly, the retail price and recycling ratio of Bayesian equilibrium are respectively:

$p^{M-N^{*}}=\frac{[6C_{L}-\beta\Delta^{2}(1-a^{2})(3-a)][\alpha_{0}+t(f-\alpha_{0})]+2C_{L}\beta c_{m}}{\beta[8C_{L}-\beta\Delta^{2}(1-a^{2})(3-a)]}$,

$\tau_{r}^{M-N^{*}}=\frac{\Delta\left(1-a^{2}\right)\left(\alpha_{0}-\beta c_{m}\right)}{8C_{L}-\beta\Delta^{2}\left(1-a^{2}\right)\left(3-a\right)}\text{+}\frac{\Delta\left(1-a^{2}\right)t\left(f-\alpha_{0}\right)}{8C_{L}-\beta\Delta^{2}\left(1-a^{2}\right)\left(3-a\right)}$. (B7)

Based on the expectation rule, the unconditional t expected profits of the manufacturer and retailer are respectively:

,

$\pi_{r}^{M-N^{*}}\text{=}\frac{C_{L}[4C_{L}-\beta\Delta^{2}(1-a^{2})(2+a)](\alpha_{0}-\beta c_{m})^{2}}{\beta[8C_{L}-\beta\Delta^{2}(1-a^{2})(3-a)]^{2}}+\frac{C_{L}kt[12C_{L}-\beta\Delta^{2}(1-a^{2})(5-2a)]}{\beta[8C_{L}-\beta\Delta^{2}(1-a^{2})(3-a)]^{2}}$. (B8)

**Model-M with Information sharing**

When retailer shares her forecast demand information, the manufacturer's expectations for the retailer's price and recycling rate are:

$E(p^{M-S})=\frac{[\alpha_{0}+t(f-\alpha_{0})+\beta w]}{2\beta}-\frac{b^{2}(1-a^{2})[\alpha_{0}+t(f-\alpha_{0})-\beta w]}{2[4C_{L}-\beta b^{2}(1-a^{2})]}$,

$E(\tau_{r}^{M-S})=\frac{b(1-a^{2})[\alpha_{0}+t(f-\alpha_{0})-\beta w]}{[4C_{L}-\beta b^{2}(1-a^{2})]}$. (B9)

The manufacturer's expected profit decision model is as follows:

$\mathop{Max}\limits_{w,\tau_{m},b} E(\pi_{m}^{M-S}\left|f\right.)=([w-c_{m}+\Delta(\tau_{m}+\tau_{r})](\alpha-\beta p)-\frac{C_{L}(\tau_{m}^{2}+a\tau_{r}^{2})}{1-a^{2}}-b\tau_{r}(\alpha-\beta p)\left|f\right.)$. (B10)

Same as Model-M without information sharing, we make $b=\Delta$. Then wholesale price and the recycling rate of the manufacturer can be expressed as:

$w^{M-S^{*}}=\frac{[4C_{L}-\beta\Delta^{2}(1-a^{2})(2-a)][\alpha_{0}\text{+}t(f-\alpha_{0})]+[4C_{L}-\beta\Delta^{2}(1-a^{2})]\beta c_{m}}{\beta[8C_{L}-\beta\Delta^{2}(1-a^{2})(3-a)]}$,

$\tau_{m}^{M-S^{*}}=\frac{\Delta(1-a^{2})[\alpha_{0}+t(f-\alpha_{0})-\beta c_{m}]}{8C_{L}-\beta\Delta^{2}(1-a^{2})(3-a)}$. (B11)

Accordingly, the retail price and recycling ratio of Bayesian equilibrium are respectively:

$p^{M-S^{*}}=\frac{[6C_{L}-\beta\Delta^{2}(1-a^{2})(3-a)][\alpha_{0}+t(f-\alpha_{0})]\alpha_{0}+2C_{L}\beta c_{m}}{\beta[8C_{L}-\beta\Delta^{2}(1-a^{2})(3-a)]}$,

$\tau_{r}^{M-S^{*}}=\frac{\Delta(1-a^{2})[\alpha_{0}+t(f-\alpha_{0})-\beta c_{m}]}{8C_{L}-\beta\Delta^{2}(1-a^{2})(3-a)}$. (B12)

Based on the expected value rule, the unconditional expected profit of the retailer and the manufacturer is respectively:

$\pi_{m}^{M-S^{*}}\text{=}\frac{C_{L}(\alpha_{0}-\beta c_{m})^{2}\text{+}C_{L}kt}{\beta[8C_{L}-\beta\Delta^{2}(1-a^{2})(3-a)]}$,

$\pi_{r}^{M-S^{*}}\text{=}\frac{C_{L}[4C_{L}-\beta\Delta^{2}(1-a^{2})(2+a)][(\alpha_{0}-\beta c_{m})^{2}\text{+}kt]}{\beta[8C_{L}-\beta\Delta^{2}(1-a^{2})(3-a)]^{2}}$. (B13)
